# Pseudocapsule-Based Resection for Pituitary Adenomas *via* the Endoscopic Endonasal Approach

**DOI:** 10.3389/fonc.2021.812468

**Published:** 2022-01-17

**Authors:** Yuefei Zhou, Jialiang Wei, Feng Feng, Jianguo Wang, Pengfei Jia, Shuangwu Yang, Dakuan Gao

**Affiliations:** ^1^ Department of Neurosurgery, Xijing Hospital, Fourth Military Medical University, Xi’an, China; ^2^ Department of Rehabilitation Medicine, Xijing Hospital, Fourth Military Medical University, Xi’an, China; ^3^ Department of Neurosurgery, Shenmu County Hospital, Yulin, China

**Keywords:** endoscopic endonasal approach, pituitary adenoma, pseudocapsule, extracapsular resection, skull base reconstruction, cerebrospinal fluid leakage

## Abstract

**Introduction:**

The endoscopic endonasal approach (EEA) is a safe and effective treatment for pituitary adenomas (PAs). Since extracapsular resection (ER) of PAs improves tumor resection and endocrine remission rates, the interface between the pseudocapsule and gland draws increasing attention. However, it is difficult to precisely dissect the tumor along the exact boundary, and complete removal of the tumor increases the risks of normal tissue damage and cerebrospinal fluid (CSF) leakage. In this study, we investigated the extracapsular resection as well as the pseudocapsule histology to evaluate the effectiveness and safety of pseudocapsule-related surgical interventions.

**Methods:**

From December 2017 to December 2019, 189 patients of PAs *via* EEA in our single center were analyzed retrospectively. The images, operative details, and clinical follow-up of patients were collected. Sixty-four patients underwent pseudocapsule-based ER, and 125 patients also underwent traditional intracapsular resection (IR) with or without intensive excision for FPAs. The clinical characteristics, tumor resection, endocrinological outcomes, and postoperative morbidities of the two groups were compared. Informed consent for publication of our article was obtained from each patient. Histological examination of pseudocapsule was performed using hematoxylin and eosin and reticulin staining.

**Results:**

The gross total recession was 62 (96.9%) in the ER group and 107 (85.6%) cases in the IR group, whereas the endocrine remission rate was 29/31 (93.5%) and 40/53 (75.5%) cases, respectively. Anterior pituitary functions were not aggravated postoperatively in any patient, but transient diabetes insipidus (DI) occurred more in the IR group (64.0%) than in ER (48.4%). Pseudocapsule specimens were obtained in 93 patients, and clusters of small cell aggregation were detected in 11 pseudocapsule specimens (11.8%) whereas other patients showed no remarkable developed pseudocapsule. Intraoperative CSF leak occurred more in the ER group (28.1%) than in the IR group (13.6%), but no difference was seen between two groups postoperatively. No case of intracranial hematoma or pituitary crisis occurred in both groups. After a mean follow-up of 22.8 months, tumor recurrence was observed in 4 (2.1%) cases.

**Conclusion:**

Pseudocapsule-based extracapsular resection of PAs *via* EEA is an effective and safe procedure to achieve complete resection with high and sustained endocrine remission and without deteriorating pituitary function.

## Introduction

Over the last two decades, the endoscopic endonasal approach (EEA) has been extensively developed and refined for the resection of pituitary adenomas (PAs). The endoscopic panoramic view is superior in terms of efficacy and safety for sellar surgery, and studies have reported that PAs can be effectively resected by EEA with minimal postoperative morbidity (1). A pseudocapsule between PA and normal adenohypophysis was initially observed by Costello in 1936 (2). Oldfield and colleagues used the phrase “surgical capsule of adenoma” to describe this histologically confirmed pseudocapsule in 2006 which was found in about 50% of patients and tends to be more frequent in larger tumors (3). The studies elaborated procedure along the outer face of the pseudocapsule between the adenoma and surrounding normal gland tissue achieved radical removal of the tumor while preserving normal pituitary function (4–6). Thus, in recent years, extracapsular resection (ER), which emphasized the importance of pseudocapsule as a surgical plane, was adopted for more radical resection of the tumor (7).

Although PAs were frequently present within the pseudocapsule and complete tumor resection using the ER technique has been reported to maximize the effectiveness for PAs with pseudocapsules (4, 8), many authors believe that resection without compromising pituitary function is imperative to improving the ultimate health outcome of patients. In some selective cases, an incomplete adenoma resection is advised because it is expected that this is best for the patients, through lower complication rates and preserving pituitary function. The actual effects of ER-based complete resection of PA are still under debate.

To explore the effectiveness and safety of the operative management of the pseudocapsule, we grouped our patients based on the resection techniques we adopted. The surgical and endocrinological outcomes and complications were also collected and analyzed to evaluate the potential benefits or flaws of ER surgery. This current manuscript reports our preliminary experience about pseudocapsule-based resection procedure with different strategies in a series.

## Materials and Methods

### General Data and Clinical Manifestations

In this retrospective study, we reviewed patients in our single institution (Department of Neurosurgery, Xijing Hospital, Fourth Military Medical University, Xi’an, China) who underwent EEA for PAs from December 2017 to December 2019. The information collected from patients’ electronic medical records included presenting symptoms, operative notes, postoperative course, histopathological diagnosis, laboratory data, and image files. Informed consent was obtained from all patients.

In the current study, patients with other primary endocrine diseases, with obviously suprasellar and parasellar extensions or with cavernous sinus invasion (Knosp 4), were excluded from this study. The average age and disease history were 48.2 years and 16.5 months, respectively. All cases were given initial surgical treatment, and tumor recurrence postoperatively was removed from the groups.

### Endocrinological Evaluations

All patients underwent a baseline preoperative pituitary hormone examination including serum cortisol, free thyroxine, thyroid stimulation hormone (TSH), adrenocorticotropic hormone (ACTH), growth hormone (GH) and insulin-like growth factor-1 (IGF-1), prolactin (PRL), luteinizing hormone (LH) and follicle-stimulating hormone (FSH), testosterone (in males), and estradiol (in females). Postoperative biochemical remission was defined as a nadir serum GH level of <0.4 ng/ml after an oral glucose load and/or a subsequently normal IGF-1 level adjusted for gender and age for acromegaly; morning serum cortisol level that was <5 μg/dl within 1 week postoperatively and thereafter, indicating no evidence of hypercortisolism for Cushing disease; normalized morning serum TSH, free triiodothyronine (FT3), and FT4 levels for thyrotroph adenoma; and serum PRL level of <15 ng/ml for prolactinoma. Diabetes insipidus (DI) was diagnosed when hypotonic polyuria was >3,000 ml/day. Hormonal status was evaluated at 1 week and 3 months after surgery and twice per year thereafter to evaluate anterior pituitary functions.

### Imaging Analysis

**Figure 1:**
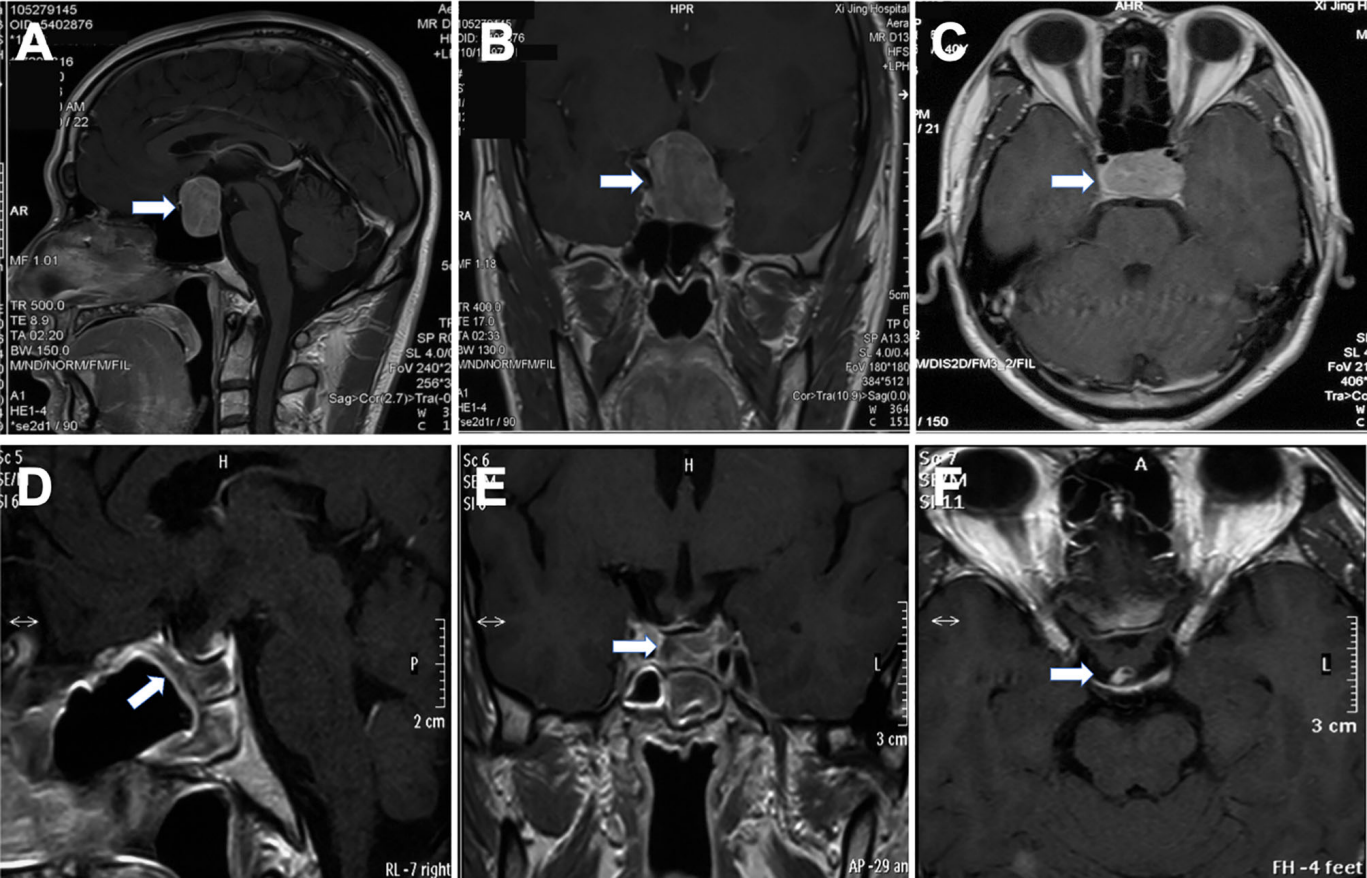
Preoperative and postoperative magnetic resonance images of pituitary adenoma. The lesion was seen in the sellar region **(A, B),** and the normal pituitary gland was squeezed to the right side (arrow) **(C)**. The tumor was excised completely by EEA, mucosal flap was in good condition **(D)**. Optic chiasm and pituitary stalk were in normal position **(E, F)**.

The imaging and volumetric assessment and analysis were independently performed by an experienced neuroradiologist with access to all imaging sequences. Computed tomography is useful for demonstrating the degree of pneumatization and locations of septations in the sphenoid sinus. The magnetic resonance imaging (MRI) scanning was performed before surgery to provide excellent details about the tumor’s size and texture and the location of normal adenohypophysis and pseudocapsule. The distribution and density of the pituitary gland could be seen on T1-weighted MR images (**Figure 1**). The position of the anterior communicating artery and internal carotid artery could be seen on T2-weighted images, also enabling us to reduce the surgical risks. The degree of resection was calculated by measuring the residual tumor volume using MRI data.

### Surgical Procedure and Technique

Under general anesthesia by endotracheal intubation, patients were in a supine position with the head rotated to the right side. A fascia lata donor site is also prepared to harvest autologous fascia for skull base reconstruction, in case of nasoseptal flap deficit. The operation proceeds with a binostril technique: one surgeon works bimanually while another one drives the endoscope to facilitate 3D perception of the surgical field. The middle turbinate was pushed laterally to enhance visibility, and a needle electrode was used to make a pedicled nasoseptal flap. A wide sphenoidotomy is important as it allows more degrees of freedom for instrument manipulation in the tumor cavity. It is also important to ensure meticulous hemostasis when operating in the nasal cavity, as it may be a constant source of blood rundown into the surgical field. Furthermore, the septations were drilled away carefully by not damaging the internal carotid artery, and the mucosa over the sella was opened in a curtain-like manner or fully removed to allow for optimal bony anatomy identification. Navigation can be used to compensate the under-pneumatized sinus for safety. To benefit from the panoramic view the endoscope offers, a larger bone window of the sellar floor is preferred and was further tailored according to preoperative MRI findings to protect the healthy pituitary gland, prevent any new endocrine deficit, and reduce the difficulty of skull base reconstruction. The bone over the carotid arteries and sellar floor was drilled with copious irrigation to avoid thermal injury to the underlying neurovascular tissues, and an eggshell bone could be made to facilitate bone resection. A rongeur is used, if necessary, to satisfactory extend the bone window, and Doppler is used to ensure security. Venous bleeding was usually easily controlled with SURGIFLO (Ethicon, America) and gentle pressure.

**Figure 2:**
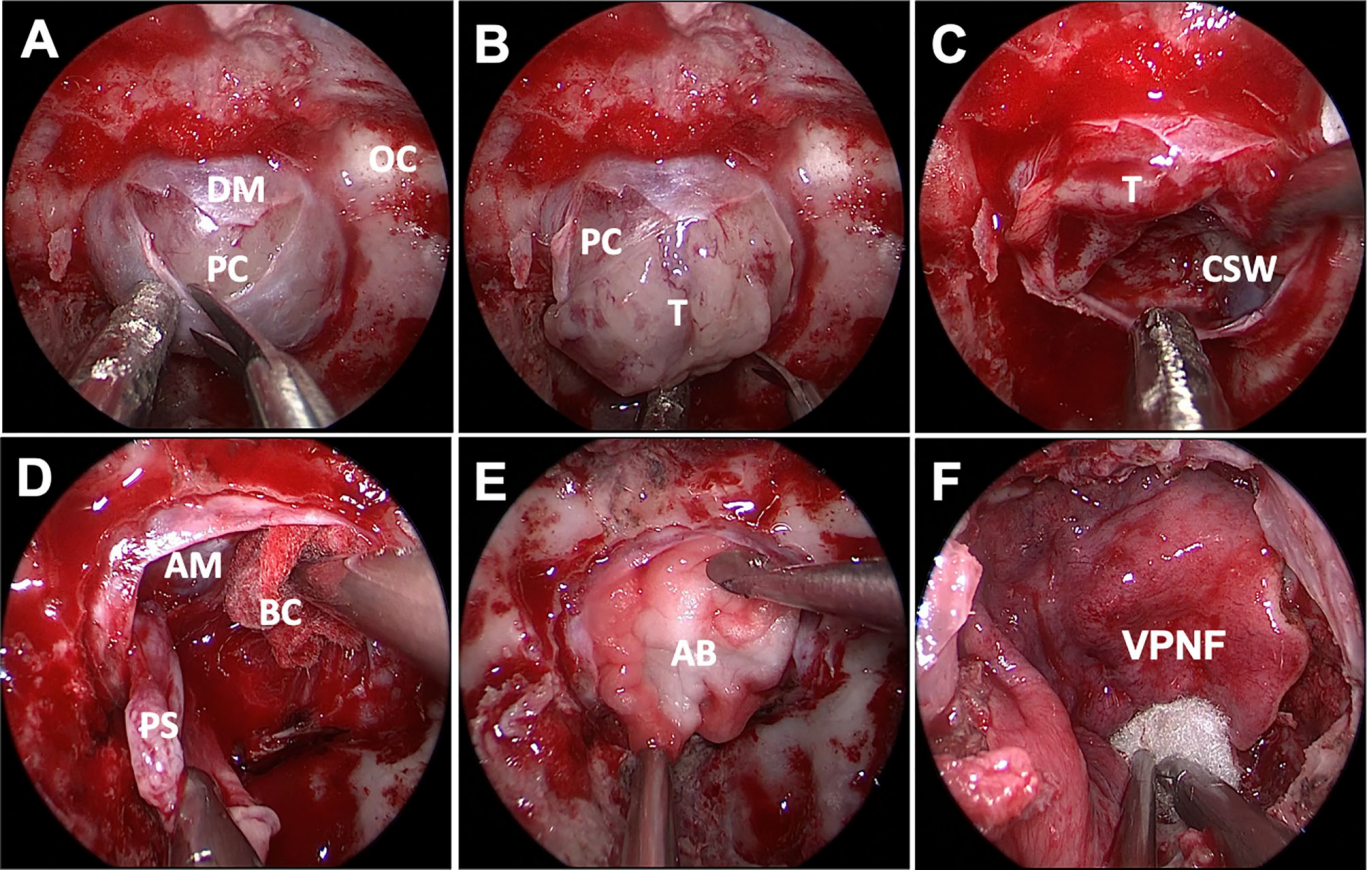
Endoscopic endonasal approach of PA surgery. The soft and gray tumor was visualized after sellar floor opening and dura incision, but no obvious pseudocapsule was found **(A, B)**. The inferior and lateral were removed to expose the cavernous sinus wall **(C).** After adequate intracapsular debulking, the pseudocapsule was carefully separated under the protection of brain cotton without hurting the arachnoid membrane of suprasellar cistern **(D)**.An absorbable artificial biomembrane was placed firstly **(E)** before the vascularized pedicled nasoseptal flap **(F)** to reconstruct the skull base. DM, dura mater; OC, optic canal; PC, pituitary capsule; T, tumor; CSW, cavernous sinus wall; AM, arachnoid membrane; BC, brain cotton; AB, artificial biomembrane; VPNF, vascularized pedicled nasoseptal flap.

The dura incision can be flexibly adjusted to meet the need of surgical procedures. Depending on different tumor sizes and pseudocapsule development, we adopted different resection strategies. 1) In microadenoma, the exposed surface of the pituitary gland looks completely normal; a small cut was made in the gland at the location where the adenoma is expected according to preoperative imaging. The right dissector was used to separate the tumor and to preserve the integrity of the pseudocapsule, and achieved total extracapsular resection. Usually, the microadenoma texture is soft, limiting the option of extracapsular dissection. With small ring curettes, the tumor is removed and the tumor cavity was explored meticulously. 2) For macroadenomas, no attempt is made to remove the entire tumor or pull it forward during the initial phases of the dissection. After the intracapsular tumor is debulked and partially removed followed by a median-lateral or basal-superior order (**Figures 2A–C**), the residual tumor was separated carefully along the pseudocapsular interface. 3) If the pseudocapsule was not visible in the first stage, we used conventional conservative intracapsular resection. Internal debulking was continued until visualization of the pseudocapsule or cavernous sinus wall was achieved (**Figure 2D**). Extrapseudocapsulary dissection was continued along the plane, preserving as much integrity of the pseudocapsule as possible. 4) After internal debulking, if the pseudocapsule was still unidentifiable, the adenoma was excised piecemeal progressively. Noteworthily, we adopted intensive excision and meticulous sweeping to remove small remnants that are hidden behind the fibrin membranes for PA. The surface of the pituitary gland was peeled off as thin a slice as possible, and the tumor bed was circumferentially resected to remove any small tumor remnant in Cushing disease or acromegaly patients. 5) To minimize the impact on pituitary functions, the suspicious tissue was sent to the pathology department for histopathology intraoperatively. A residual fragmental pseudocapsule may be beneficial if further dissection increases the risk of unacceptable neurological morbidity or obvious cerebrospinal fluid (CSF) leakage.

After tumor resection, the surgical areas were repeatedly irrigated with warm saline to reduce potential inflammation and to achieve hemostasis. It can be useful to fill the resection cavity with saline and dive into the cavity with the endoscope, rinsing continuously for detailed inspection. Depending on the presence or absence of CSF leakage during surgery, we also adopted different strategies for skull base reconstruction. 1) If no CSF leak was observed intraoperatively, the reconstruction is simple and standardized. The sphenoidal mucosa is also draped over the sella to promote remucosalization for microadenoma. The nasoseptal flap can also be restored and located in its original position. 2) The arachnoidal tear was closed by placing a small piece of abdominal fat or gelatin sponge, and overpack should be avoided. The free nasoseptal graft that was harvested at the beginning of the surgery is placed on the sellar floor. 3) In patients with bigger CSF leakage, an absorbable artificial biomembrane supported with fat or gelatin sponge was vital to preventing CSF leaks. Furthermore, pedicled nasoseptal flap was covered on the sellar floor for further reinforcement (**Figures 2E, F**). Surgicel and gelatin sponges were stuffed around to further enhance the flap in case of displacement or migration, and to accelerate healing. 4) The anterior sellar dura could also be reconstructed by dural suturing. 5) If the mucosal flap is defective, the fascia lata can further strengthen the skull base. Lumbar drainage was not used as a preventive maneuver postoperatively.

### Pathological Examinations

All resected tumor tissues were evaluated by routine pathological and immunohistochemical examination. The composition of complete and fragmentary pseudocapsules was pathologically examined. All tissues obtained in the study were paraformaldehyde fixed and paraffin embedded. The sections were stained using hematoxylin and eosin staining or Masson’s trichrome staining.

### Statistical Analysis

Statistical analyses were conducted by blinded researchers. All values of each group were presented as means ± SD. Statistical difference was analyzed, according to different comparison situations, by the chi-square test with Fisher’s exact test. *p* < 0.05 was considered statistically significant.

## Results

Sixty-four patients received ER surgery, and 125 patients received IR surgery. The follow-up was conducted 1 and 6 months postoperatively and annually thereafter. The overall mean follow-up period was 6∼42 months (average 22.8 months). According to preoperative MRI findings, the tumors consisted of 56 microadenomas (<10 mm) and 133 macroadenomas (≥10 mm); the overall mean value of the tumor diameter was 22.4 mm. There were statistically significant differences in age (*p* = 0.0228) and maximum tumor diameter (*p* = 0.0007) between the two groups, respectively (**Table 1**).

**Table 1:** Preoperative characteristics of patients.

Symptoms preoperative (%)	Total (n = 189)	Extracapsular resection (n = 64)	Intracapsular resection (n = 125)	p value
***Male***	90 (47.6%)	31 (48.4%)	59 (47.2%)	0.8719*^b^*
***Age***	48.2 ± 11.0	51.7 ± 12.9	47.1 ± 13.1	0.0228*^a^*
***Visual impairment***	86 (45.5%)	28 (43.8%)	58 (46.4%)	0.7292*^b^*
***Functional adenoma***	84 (44.4%)	31 (48.4%)	53 (42.4%)	0.4292*^b^*
***Acromegaly***	31 (16.4%)	14 (21.9%)	17 (13.6%)	0.1460*^b^*
***Prolactinoma***	45 (23.8%)	13 (20.3%)	32 (25.6%)	0.4193*^b^*
***Cushing disease***	5 (2.6%)	2 (3.1%)	3 (2.4%)	0.7688*^b^*
***Thyroid dysfunction***	3 (1.6%)	2 (3.1%)	1 (0.8%)	0.2262*^b^*
***Maximum tumor diameter (mm)***	22.4 ± 7.9	20.3 ± 6.2	24.5 ± 8.7	0.0007*^a^*
***Tumor size***				
***Microadenoma (<10 mm)***	56 (53.1%)	34 (53.1%)	22 (17.6%)	<0.0001*^b^*
***Macroadenoma (≥10 mm)***	133 (23.8%)	30 (46.8%)	103 (82.4%)	<0.0001*^b^*

^a^The Student t test was used for statistic analysis.

^b^The chi-square test was used for statistic analysis.

### Tumor Removal

**Figure 3:**
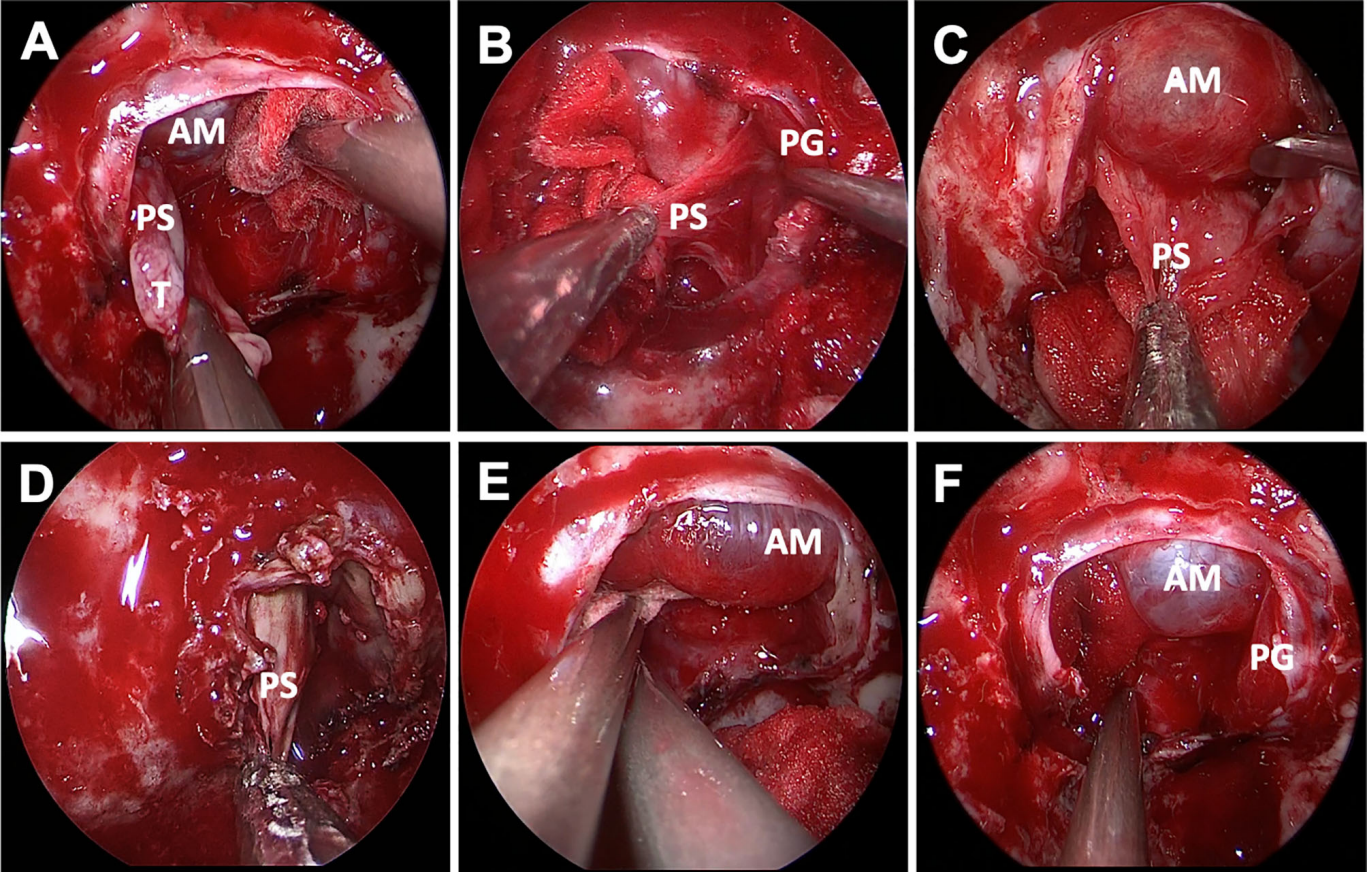
Endoscopic endonasal view of pseudocapsular excision. The distinct pseudocapsule during surgery was exposed **(A)**. Relative interface between the pituitary adenoma and the normal pituitary gland was observed after adequate intracapsular debulking, and only degenerated pituitary tissue or fibers were detected by pathological examination **(B)**. The pseudocapsule adhered tightly to the arachnoid membrane and was separated using a dissector **(C)**. The pseudocapsule was firm and dissected piece by piece **(D)**. Bleeding from the cavernous sinus was packed with gelatin sponge, and the normal pituitary gland can be seen without arachnoid membrane injury after tumor resection **(E, F)**. AM, arachnoid membrane; PS, pseudocapsule; PG, pituitary gland; PP, posterior pituitary; GS, gelatin sponge.

Postoperative MRI showed gross total resection achieved in 62 (96.9%) in the ER group and 107 (85.6%) cases in the IR group. There was no statistical difference in the incidence of recurrence between two groups (*p* = 0.900) (**Table 2**). Among 64 patients in the ER group, the pseudocapsule was apparent and complete enough to be used as a surgical plane initially in only 27 patients; for the remaining 37 patients, the pseudocapsule was identified at the outer margin of the tumor after internal debulking. The tumors were removed in a piecemeal fashion in 125 patients (66.1%) whose pseudocapsule was not well-demarcated and fragmentized (**Figure 3**).

**Table 2:** Postoperative characteristics of patients.

Symptoms postoperative	Total (n = 189)	Extracapsular resection (n = 64)	Intracapsular resection (n = 125)	p value
***Gross total resection***	169 (89.4%)	62 (96.9%)	107 (85.6%)	**0.0171**
***Tumor recurrence***	4 (2.1%)	1 (1.6%)	3 (2.4%)	0.7050
***Intraoperative CSF leakage***	35 (18.5%)	18 (28.1%)	17 (13.6%)	**0.0150**
***postoperative CSF rhinorrhea***	1 (1.6%)	0	1 (1.6%)	0.4731
***Transient diabetes insipidus^b^***	111 (58.7%)	31 (48.4%)	80 (64.0%)	**0.0397**
***Endocrinological remission [n (%)]^a^***	69/84 (82.1%)	29/31 (93.5%)	40/53 (75.5%)	**0.0369**

^a^Postoperative endocrinological remission was defined as: a nadir serum GH level of < 0.4 ng/ml after an oral glucose load and/or a subsequently normal IGF-1 level adjusted for gender and age for acromegaly; morning serum cortisol level that was >5 μg/dl within 1 week postoperatively for Cushing disease; normalized morning serum TSH, free triiodothyronine (FT3) and FT4 levels for thyrotroph adenoma, and serum PRL level of <15 ng/ml for prolactinoma.

^b^Diabetes insipidus (DI) was diagnosed as hypotonic polyuria > 3,000 ml/day.

The chi-square test was used for statistic analysis. Bold formation means the differences that reached the statistically significance.

Pseudocapsules were identified in 48.4% of functioning pituitary tumors and in 51.6% of non-functioning ones. Considering the postoperative risk of CSF leak, we fragmentalized the pseudocapsule and reserved part of the pseudocapsule that is adjacent to the suprasellar cistern arachnoid in 23/125 (18.4%) patients. Excessive resection of the cavernous sinus wall resulted in abducens nerve palsy in 1 case, which returned to normal after a 2-month treatment. A larger portion of microadenoma patients received ER surgery, compared with the IR group (*p* < 0.0001) (**Table 2**).

### Endocrine Outcome

Pituitary function was tested 3 months postoperatively and at regular intervals on an individual basis depending on the patient’s clinical status. Overall, the remission rate was higher in the ER group (93.5%, 29/31) than in the IR group (75.5%, 40/53) (*p* = 0.042). In addition, anterior pituitary functions were not aggravated in any patient postoperatively. Transient DI was a more common symptom in the IR group (80 in IR vs. 31 in ER, *p* = 0.044). One acromegalic patient in the ER group, who had achieved early biochemical remission by EEA alone, showed elevated serum IGF-1 levels at 1 year postoperatively. The patient received octreotide acetate microspheres (20 mg) for injection and achieved endocrinological relief after 3 months (**Table 2**).

### Histopathology Features

Surgically resected specimens were examined histologically. All preoperative clinical diagnoses were confirmed by pathological examination. The classification of PAs according to hormonal activity was verified from the postoperative immunohistochemistry of the tumor tissue. Endocrine-active tumors were observed in 84 patients (44.4%); PRL, GH, ACTH, and TSH-secreting tumors were observed in 45, 31, 5, and 3, respectively, and non-functioning tumors were confirmed in 105 patients (55.6%). There was no difference between groups (*p* = 0.444).

**Figure 4:**
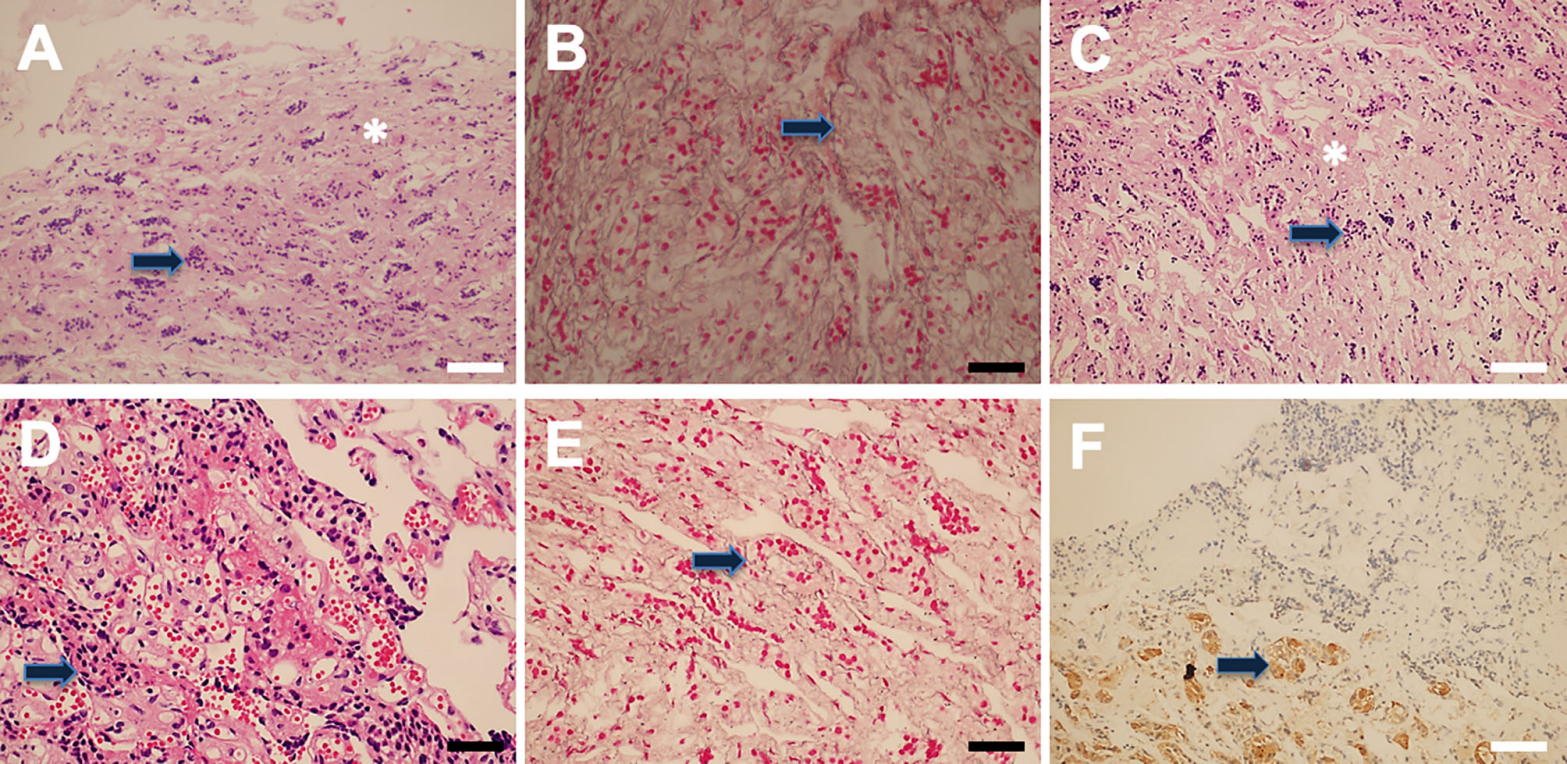
Pseudocapsule histopathology. The pituitary-like tissue (arrow) and interfibrillar substance (asterisk) were found **(A)**. Reticulin staining results showed normal pituitary tissue(arrow) **(B)**. Hematoxylin and eosin (H&E) staining showed normal adenohypophyseal components in the fibrous capsule, which exhibited acinar-like morphology **(C, D).** Reticulin staining showed an intact mesh structure inside the fibrous capsule **(E),** and PRL immune-reactivity was also detected **(F)**. White bar = 100 μm, black bar = 50 μm.

**Figure 5:**
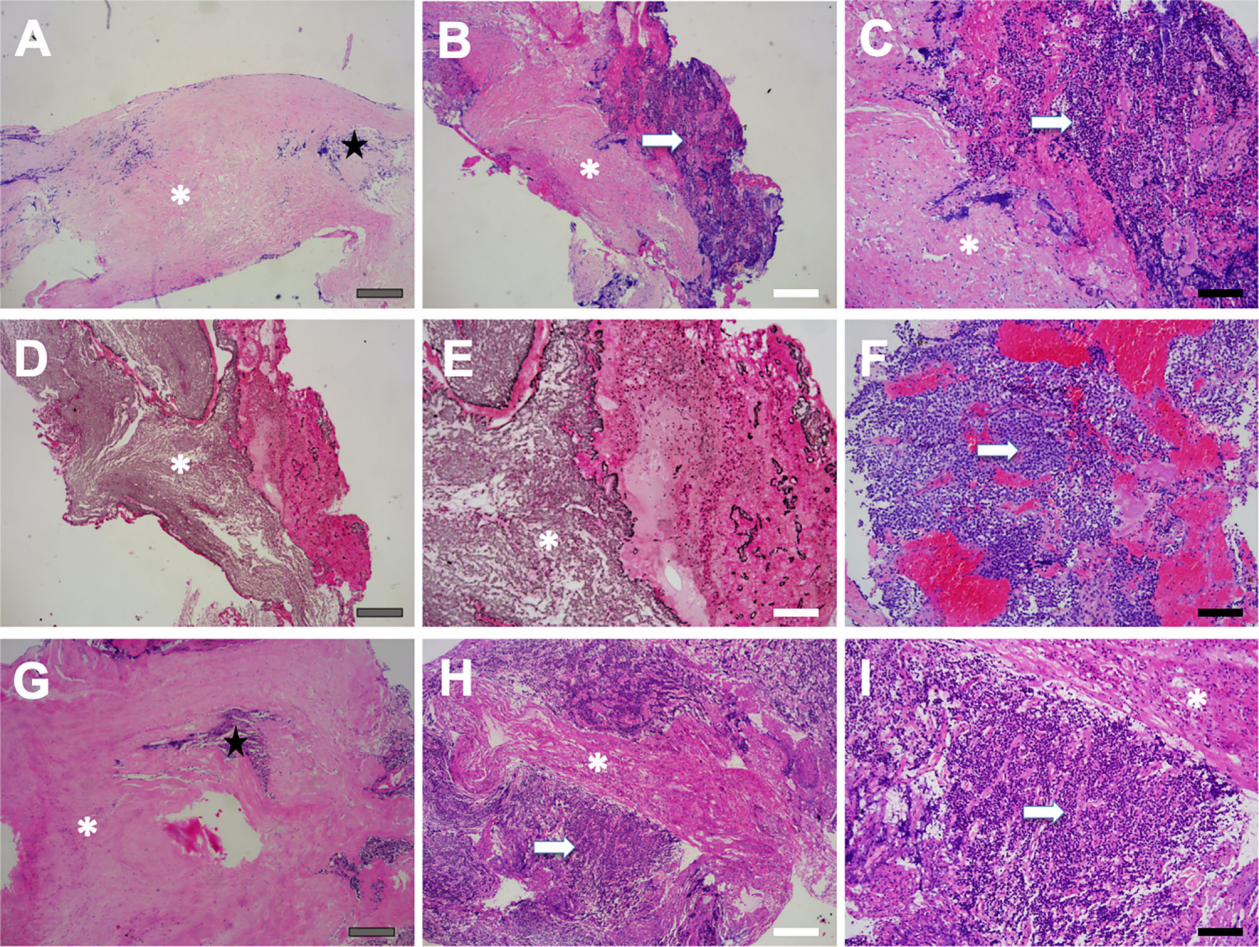
Pseudocapsule specimen histopathology. Patchy fibrous tissue (asterisk) as well as a few pituitary-like cells (star) were found **(A)**, and the fibrous tissue was surrounded by pituitary-like cells **(B, C)**. Reticulin staining showed destruction and disorder of adenoid structure of hyperplastic pituitary-like cells, suggesting the pituitary adenoma **(D, E)**. Tumor cells were exhibited as small cell aggregation, which was found in pseudocapsule specimen (arrow) **(F)**. The pseudocapsule contained normal pituitary tissue (star) **(G)**. Pituitary adenoma (arrow) featured with homo-size tumor cells as well as vascularized structure, and fibrous hyperplasia was also observed (asterisk) **(H, I)**. Gray bar = 200 μm, white bar = 100 μm, black bar = 50 μm.

Pseudocapsular specimens were histologically examined using hematoxylin and eosin and reticulin staining in 93 (49.2%) samples. The results revealed that the pseudocapsules were composed of fibroblasts, collagen fibers, and condensations of small cells on the background of myxoid materials (**Figure 4**). Reticulin staining demonstrated that the pseudocapsule existed in the adjacent pituitary even in patients without visible pseudocapsule intraoperatively (**Figure 5**).

### Cerebrospinal Fluid Leak and Efficiency of Skull Base Construction

Intraoperative CSF leakage rates of the ER and IR groups were 28.1% (18/64) and 13.6% (17/125), respectively (*p* = 0.018). However, no difference was seen in postoperative CSF leakage of two groups (*p* = 0.773). One case in the ER group developed CSF rhinorrhea with pulmonary infection postoperatively and was cured after reoperation (**Table 2**).

## Discussion

The pseudocapsule was first described in the early 1900s, which was formed by the compression between the tumor and normal gland (3). Adenoma growth leads to compression of the acinar structure of the adjacent normal gland, resulting in a reticulin-rich pseudocapsule that encases the entire adenoma in. Since Oldfield and Vortmeyer firstly dissected the pseudocapsule around microadenomas instead of internal piecemeal removal (3, 6), the understanding of pseudocapsule formation and clinical significance has been developed. Early identification of the adenoma edges is crucial to limiting the risk of damage to the pituitary gland and tearing of the arachnoid at the level of the diaphragm sellae; thus, using the pseudocapsule as a surgical plane could be effective for surgeons to better confine the resection area (9).

Studies have demonstrated that the possession rate of a pseudocapsule varies depending on the characteristics of the PA, including the pathological type and tumor size (4). Whether the resection is performed outside or inside the pseudocapsule largely depends on the consistency of the tumor and the degree of pseudocapsule development. Thus, different surgical techniques should be adopted according to the presence of the resectable pseudocapsule. When a tumor has a fibrous and thick pseudocapsule or when a tumor is very hard or fibrous, dissection should be performed completely outside the pseudocapsule. Kawamata (10) suggested that, in smaller tumors, the pseudocapsule tended to exist more prominently and to cover the whole tumor, whereas in larger tumors the pseudocapsule tended to be discontinuous or disrupted. Similarly, in the present study, we found that ER was more performed in microadenomas, whereas IR was more adopted in macroadenomas. Furthermore, in some macroadenomas, the pseudocapsule could not be seen until proper intracapsular debulking. By contrast, some PAs exhibited no or undefinable pseudocapsule; during the entire procedure, the adenoma was excised piecemeal progressively with a dissector, blunt ring curette, and aspirator.

Previous studies showed that the ER helps in achieving complete tumor resection (7, 11, 12). Similarly, in the present study, patients who received ER surgery achieved a higher rate of total adenoma resection, which indicated the beneficial role of ER surgery. Studies revealed heterogeneous conclusions that there still existed a discrepancy of whether ER brings a higher chance of damage than traditional ones. Some believed that ER methods could also increase the chance of pituitary gland damage, which leads to pituitary dysfunction. Others, on the contrary, summarized optimistic results (13). Typically, since repeated PA surgery has been proved safe for the experienced doctors and well tolerated by patients, intact pituitary gland function is deemed more important than adenoma total removal (14), Theoretically, it is hard for surgeons to extirpate only tumor cells completely during surgery without removing any normal pituitary gland tissue because in most cases the adenoma directly contacts with the normal pituitary gland. Thus, although we might resect a thin layer of anterior pituitary gland when resecting the tumor using the ER method, a meticulous operation could avoid extra collateral damage of the pituitary gland and do no more harm than the traditional IR way. Besides, intracapsular resection sometimes requires more aggressive resection that could injure the remaining pituitary gland, which consequently increases the risk of worsening of pituitary function and increases the possibility of complications (15). This may be the reason why patients who received ER operation in our study showed less incidence of DI and better endocrinological remission rate after surgery, indicating that ER could cause less pituitary gland damage, compared with IR methods.

Some scholars found that the capsule itself contains tumor cells and may be a main cause of persistent hypersecretion of the hormone and possibly the source of recurrence (16). In addition, some studies found that the pseudocapsule is disrupted by tumor invasion so that the extracapsular removal and management of tumor invasion outside of the pseudocapsule are crucial to accomplishing complete PA removal (17, 18). Noteworthily, in our study, the histological staining revealed that the tumor was partially infiltrated in the pseudocapsule. Hence, if necessary, we applied intensive excision to remove all the confinable pseudocapsule and small remnants that are hidden behind the membranes to reach complete resection. New classification recognizes some subtypes of PAs as high-risk PAs, which include sparsely granulated somatotroph adenoma, lactotroph adenoma in men, silent corticotroph adenoma, and plurihormonal Pit-1-positive adenoma (19). For these refractory pituitary adenomas, we recommend aggressive resection, especially in IR resection cases. Partial gland resection or resection of the cavernous sinus medial wall is necessary in some cases since studies showed that it could help improve biochemical remission for the pituitary gland (20, 21).

Postoperative CSF leaks represent one of dreaded surgical complications encountered after EEA and have been reported to occur in approximately 3% at high-volume institutions (22). After the pedicled nasoseptal flap development by Haddad, there is a striking decrease of CSF leak rates in endonasal approaches series. The pseudocapsule resection might violate and tear the arachnoid membrane in cases that the pseudocapsule was very adhesive to the arachnoid membrane, and sometimes CSF leakage is encountered. There are many techniques to avoid CSF leaks (23); focuses on limiting trauma to the diaphragm arachnoid membrane may be the most critical factor. According to the severity of intraoperative cerebrospinal fluid leak, proper closure techniques were used. In our present study, there was just one case in CSF rhinorrhea postoperatively in the ER group due to the defective nasoseptal and adopted reoperation and revision using autologous fascia grafts. The results indicated that our skull base reconstruction is also effective for patients receiving ER surgery. Considering the heterogeneity of PA patients, we suggested personalized decision in complicated cases, and pseudocapsule remnant is acceptable if the complete removal could cause refractory CSF leak.

### Study Limitations

There is great heterogeneity in the operative procedure of PA across different institutions, with limited evidence regarding the comparative surgical tips and complications. There is a striking variance between centers for Pas (32862300), depending on local experience, alternative strategies, and so forth. There are also some differences in distinguishing the histology of pseudocapsule samples. More detection methods will be used to further observe the characteristic of the pseudocapsule. Future transmission electron microscopy is needed to further inquire the histological features of the pseudocapsule. Further data including long-term pituitary function in different groups are needed to justify the proposed techniques.

## Conclusion

Extra-pseudocapsular resection is an effective technique that maximizes the extent of resection, improves endocrinological remission, and reduces certain complication incidences.

## Data Availability Statement

The original contributions presented in the study are included in the article/supplementary material. Further inquiries can be directed to the corresponding author.

## Ethics Statement

The studies involving human participants were reviewed and approved by the Ethics Committee of Fourth Military Medical University. The patients/participants provided their written informed consent to participate in this study.

## Author Contributions

YZ and DG conceived and designed the analysis. YZ, JW, and FF wrote the manuscript. JLW analyzed the relevant data. JGW, PJ, and SY collected the MR images. All authors contributed to the article and approved the submitted version. All authors contributed to the article and approved the submitted version.

## Funding

This research was supported by the National Natural Science Foundation of China, No.81971227 and Key Project of Social Development of Shaanxi Province, No.2018ZDXM-SF-086.

## Conflict of Interest

The authors declare that the research was conducted in the absence of any commercial or financial relationships that could be construed as a potential conflict of interest.

## Publisher’s Note

All claims expressed in this article are solely those of the authors and do not necessarily represent those of their affiliated organizations, or those of the publisher, the editors and the reviewers. Any product that may be evaluated in this article, or claim that may be made by its manufacturer, is not guaranteed or endorsed by the publisher.
